# First identification and isolation of equine herpesvirus type 1 in aborted fetal lung tissues of donkeys

**DOI:** 10.1186/s12985-024-02390-2

**Published:** 2024-05-27

**Authors:** Panpan Tong, Juanjuan Pan, Yueyi Dang, Enhui Yang, Chenyang Jia, Ruli Duan, Shuyao Tian, Nuerlan Palidan, Ling Kuang, Chuanfeng Wang, Gang Lu, Jinxin Xie

**Affiliations:** 1 Laboratory of Animal Etiology and Epidemiology, College of Veterinary Medicine, Xinjiang Agricultural University, Urumqi, Xinjiang China; 2 Key laboratory for animal disease detection, College of Animal Sciences, Yili Vocational and Technical College, Yili, Xinjiang China; 3 College of Veterinary Medicine, South China Agricultural University, Guangzhou, Guangdong China; 4 Xinjiang Key Laboratory of New Drug Study and Creation for Herbivorous Animals, Urumqi, Xinjiang China

**Keywords:** Donkey, Abortion, Equid herpesvirus type 1, Neuropathogenicity, VIII group

## Abstract

**Background:**

Equine herpesvirus type 1 (EHV-1) is commonly associated with horse abortion. Currently, there are no reported cases of abortion resulting from EHV-1 infection in donkeys.

**Results:**

This was the first survey-based study of Chinese donkeys. The presence of EHV-1 was identified by PCR. This survey was conducted in Chabuchar County, North Xinjiang, China, in 2020. A donkey EHV-1 strain (Chabuchar/2020) was successfully isolated in MDBK cells. Seventy-two of 100 donkey sera were able to neutralize the isolated EHV-1. Moreover, the ORF33 sequence of the donkey-origin EHV-1 Chabuchar/2020 strain showed high levels of similarity in both its nucleotide (99.7‒100%) and amino acid (99.5‒100%) sequences, with those of horse EHV-1 strains. EHV-1 Chabuchar/2020 showed significant consistency and was classified within cluster 1 of horse EHV-1 strains. Further, analysis of the expected ORF30 nucleotide sequence revealed that donkey EHV-1 strains contained guanine at position 2254, resulting in a change to aspartic acid at position 752 of the viral DNA polymerase. Therefore, these strains were classified as horse neuropathogenic strains. Lastly, a phylogenetic tree was constructed using the partial ORF68 nucleotide sequences, showing that the identified donkey EHV-1 strain and the EHV-1 strain found in aborted Yili horses in China comprised a novel independent VIII group.

**Conclusion:**

This study showed the first isolation and identification of EHV-1 as an etiological agent of abortions in donkeys. Further analysis of the ORF33, ORF30, and ORF68 sequences indicated that the donkey EHV-1 contained the neuropathogenic genotype of strains in the VIII group. It is thus important to be aware of EHV-1 infection in the donkey population, even though the virus has only been identified in donkey abortions in China.

**Supplementary Information:**

The online version contains supplementary material available at 10.1186/s12985-024-02390-2.

## Introduction

Equine herpesvirus type 1 belongs to the family *Herpesviridae*, subfamily *Alphaherpesvirinae*, and genus *Varicellovirus* [1]. It is a highly damaging viral pathogen in horses due to its frequent association with respiratory complications, abortion, and ocular and neurological diseases. It has a substantial influence on the global equine industry as well as on animal health [2–8]. Currently, there are no documented cases of abortion resulting from EHV-1 in donkeys. However, two studies have found evidence of this virus in nasopharyngeal swabs of both symptomatic and asymptomatic donkeys with respiratory disease [9, 10].

The EHV-1 genome is composed of 150 kbp of double-stranded DNA encoding for ≥ 80 open reading frames (ORFs) [11–13]. One of these genes, ORF33, which encodes the envelope glycoprotein B (gB), contains a conserved segment that is widely used in PCR and phylogenetic analyses [14]. Similarly, the ORF30-encoded viral DNA polymerase displays a strong, but not unique, correlation with non-neuropathogenic and neuropathogenic EHV-1 strains, depending on the presence of a G or A nucleotide at position 2254. This results in the substitution of an aspartic acid (D) or asparagine (N) at position 752 of the viral DNA polymerase [8, 14–27]. Moreover, EHV-1 is classified into VII groups according to the phylogenetic analysis of ORF68 genes. These groups are associated with the geographical origin of the different EHV-1 strains [14, 16, 28].

In November 2020, a total of 80 donkeys from a farm located in Chabuchar County of Yili region (Northern Xinjiang, China), were reported to have experienced spontaneous abortions without showing any clinical symptoms. Out of a total population of 700 milk donkeys, 400 were jackasses (≥ 4 years) and 200 of 300 were pregnant mares (≥ 4 years) at an estimated seven months of gestation. This farm was established in 2019, and all the milk donkeys were brought in from other farms in the Yili area of Northern Xinjiang without any scheduled vaccination.

This is the first study conducted on EHV-1 as a causative agent of abortion in donkeys. Importantly, this is the first report on the identification, examination, and isolation of EHV-1 associated with equine abortion.

## Methods

### Sample collection

The veterinarian on the farm provided lung tissues from three of 80 aborted fetuses, and 100 sera from donkeys that had experienced abortions. Each sample of lung tissue was placed in a tube containing 1.5 mL of phosphate buffer and stored at -80 °C by the farm’s veterinarian, according to approved procedures.

### EHV detection

Viral nucleic acids were extracted (Geneaid Biotech Co.) from lung tissues, as previously described [8]. Based on previous studies on the abortions of Yili mares and donkeys in China [7, 8, 29], PCR was conducted using TIANSeq HiFi Amplification Mix (Tiangen Biotech) to detect the presence of Equine herpesvirus type 8 (EHV-8), EHV-1, EHV-4, EHV-2, and EHV-5. The primers used for the PCR analysis are listed in Table S1. The PCR protocol comprised an initial denaturation (94 °C for 2 min), proceeded by 35 cycles, each consisting of denaturation (98 °C for 10 s), annealing, and extension (68 °C for 30 s) steps. Lastly, the final extension was performed (68 °C for 5 min).

### Isolation, screening, electron microscopy, and neutralization assay of EHV-1

MDBK cells were used for the in vitro isolation of EHV-1, as reported previously [7]. Briefly, PCR-positive samples were centrifuged for 3 min at 12,000 x g, after which supernatants were passed through a 0.22 μm filter, and then used to infect MDBK cells (2 h at 37 °C in a 5% CO_2_ incubator), after which the inoculum was discarded and replaced with DMEM supplemented with 2% fetal bovine serum (FBS). The cells were then maintained for 72 h, followed by three rounds of freezing and thawing to harvest the viruses, followed by repeated inoculation for nine cell passages. Cytopathic effect (CPE) testing was then performed daily following inoculation, and the full-length OFR33, partial ORF30, and partial ORF68 sequences of EHV-1 were amplified using the primers provided in Table S2. Positive PCR amplicons were sequenced as reported previously [8]. For electron microscopy, EHV-1 particles were purified by sucrose density gradient centrifugation, as previously described [7], and were negatively stained with 2% phosphotungstic acid.

Virus neutralization tests were performed in 96-well plates. One hundred sera from donkeys with abortion were heat-inactivated by incubation at 56℃ for 30 min. The serum samples were diluted to 1:10, 1:20, or 1:40, after which an equal volume of virus stock was added and incubated at 37℃ for 60 min in a 5% CO_2_ incubator. Diluted horse anti-EHV-1 serum or serum samples from healthy horses were used as controls. After incubation, 200 µl mixtures were inoculated onto a monolayer of MDBK cells in a 96-well plate for 2 h. Each serum specimen was assessed in triplicate. After removal of the supernatant, the plate was washed twice with DMEM, and the cells were incubated with DMEM supplemented with 2% FBS for three days, after which they were checked for CPE.

### Multiple sequence alignment and phylogenetic analyses

Additional information, including the GenBank accession numbers, for the sequences in this study is provided in Figs. 3 and 4, and 5. All full-length ORF33, partial ORF30, and partial ORF68 EHV-1 nucleotide sequences isolated in this study have been submitted to GenBank, with the respective accession numbers ON584564, ON624152, and ON624153. MegAlign software was used to analyze the sequences in Lasergene v7.1, and a maximum-likelihood phylogenetic tree of all target sequences was constructed in MEGA7 with the Tamura-Nei model. The topological accuracy of the tree was assessed with 1,000 bootstrap replicates [30].

**Fig. 1:**
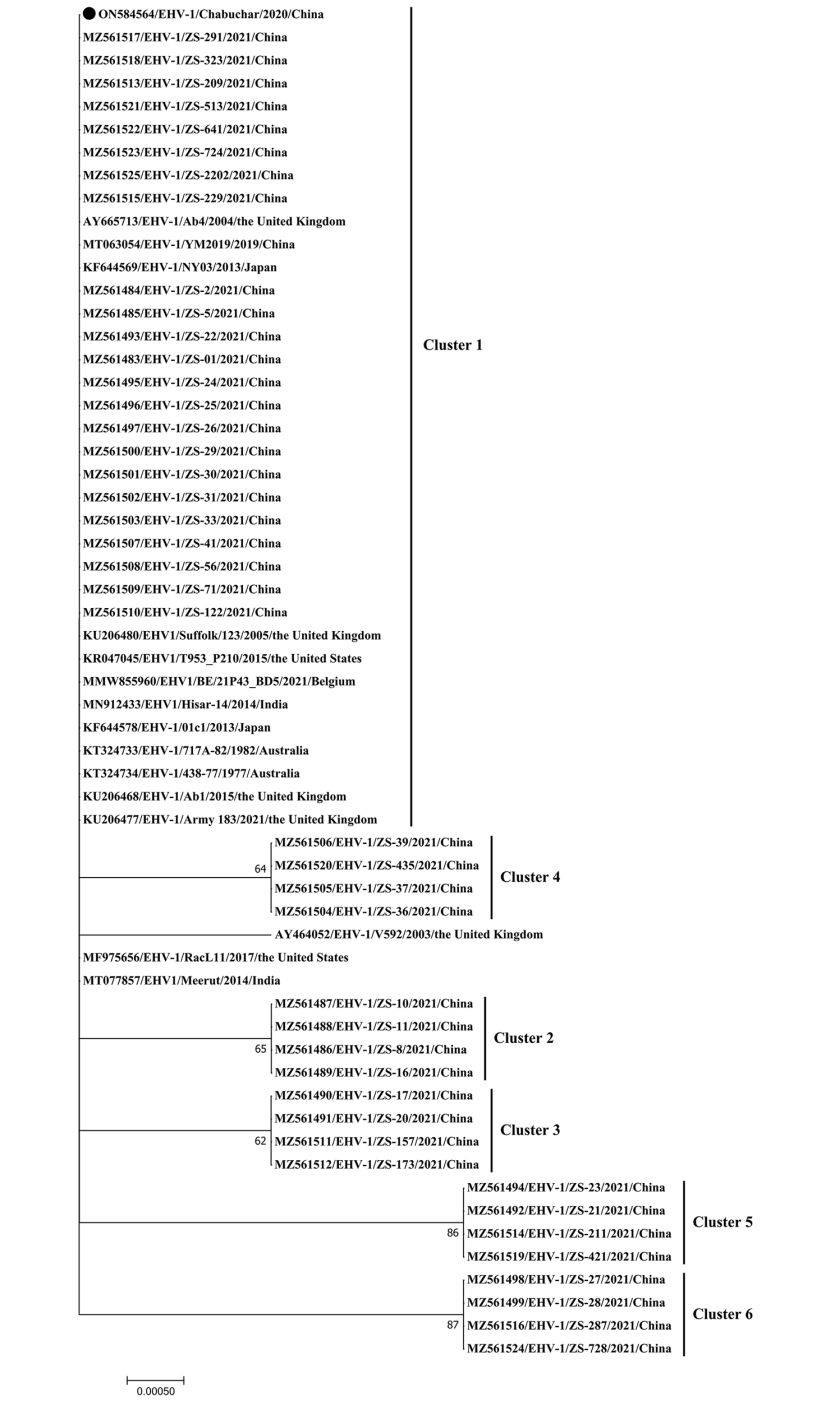
Maximum-likelihood phylogenetic tree of EHV-1 ORF33 nucleotide sequences, constructed using the Tamura-Nei model (MEGA7). The identified EHV-1 Chabuchar/2020 strain is indicated by the black circle

**Fig. 2:**
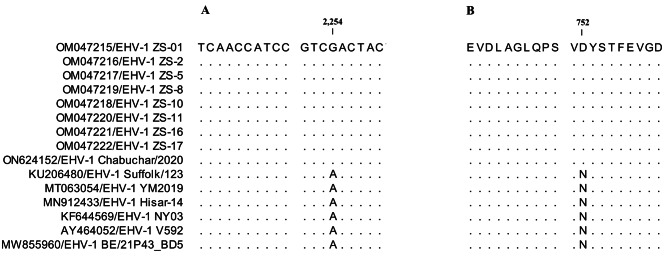
Analysis of the ORF30 gene sequence. All EHV-1 strains examined in this study displayed the G2254 nucleotide polymorphism, as shown in Fig. 4A. The Asp752 polymorphism in the EHV-1 is shown in Fig. 4B. Sequence identity with the EHV-1 ZS-01 isolate (CLC Sequence Viewer 8) is indicated by dots

**Fig. 3:**
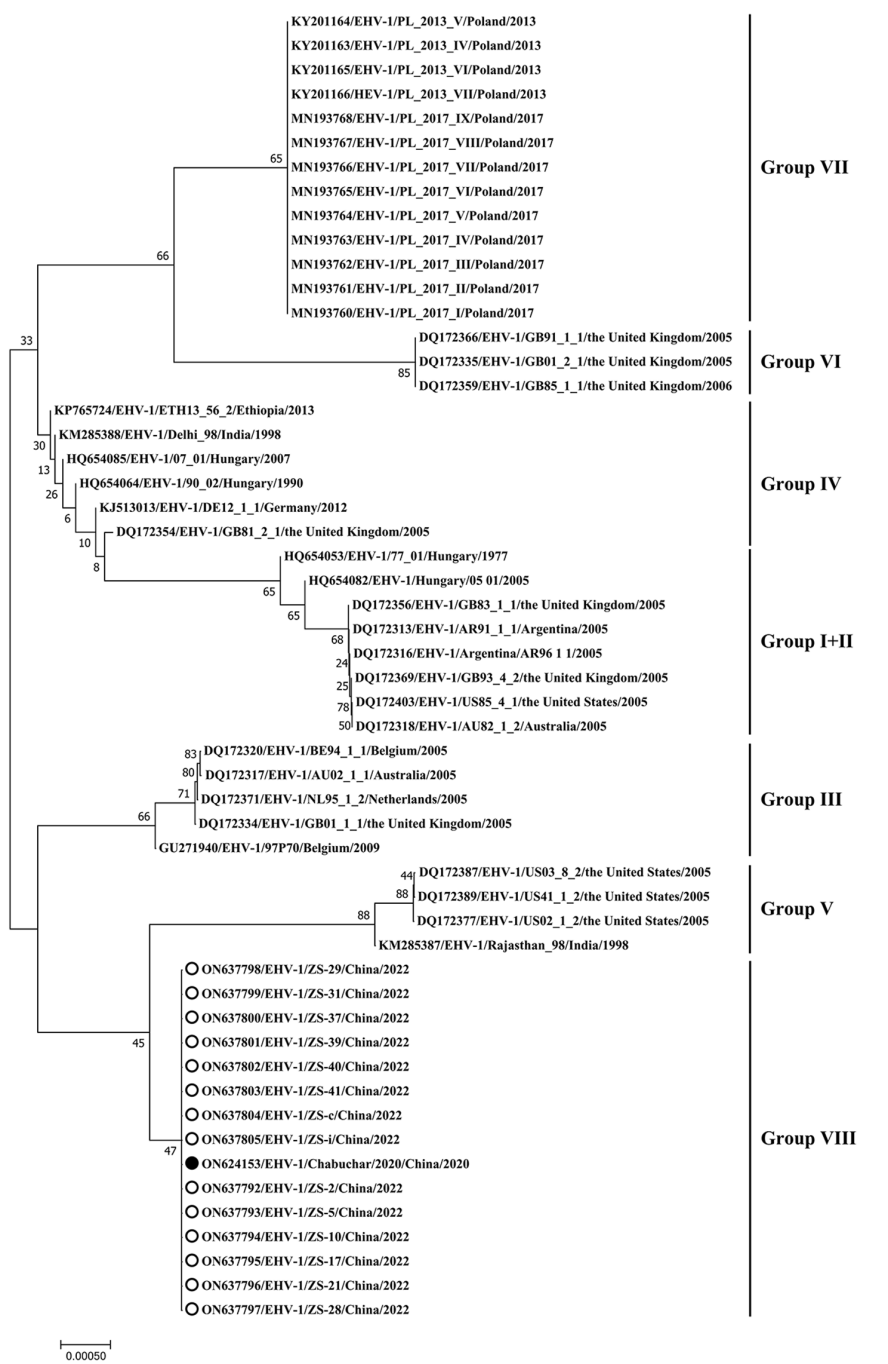
Neighbor-joining phylogenetic tree of EHV-1 ORF68 nucleotide sequences, constructed using MEGA7. The filled circle represents the newly identified EHV-1 Chabuchar/2020, while the open circles indicate EHV-1 from Yili mares

## Results

The veterinarian on the farm provided lung tissues from three aborted fetuses for the identification of the viral pathogens potentially responsible for the abortions. The PCR results confirmed the presence of EHV-1 in the lung tissues of the aborted fetuses, with a percentage positivity of 100% (3/3) (ON809533-ON809535). These results, therefore, suggested that EHV-1 could have been the cause of the abortions in the donkeys.

The EHV-1 from aborted fetal lung tissue was isolated from MDBK cells. Nine passages after inoculation, the cytopathic impact was visible (Fig. 1). The identity of the viral isolate (Chabuchar/2020 strain) was verified using transmission electron microscopy (TEM) and whole ORF33 gene sequencing (accession no: ON584564) (Fig. 2).

**Fig. 4:**
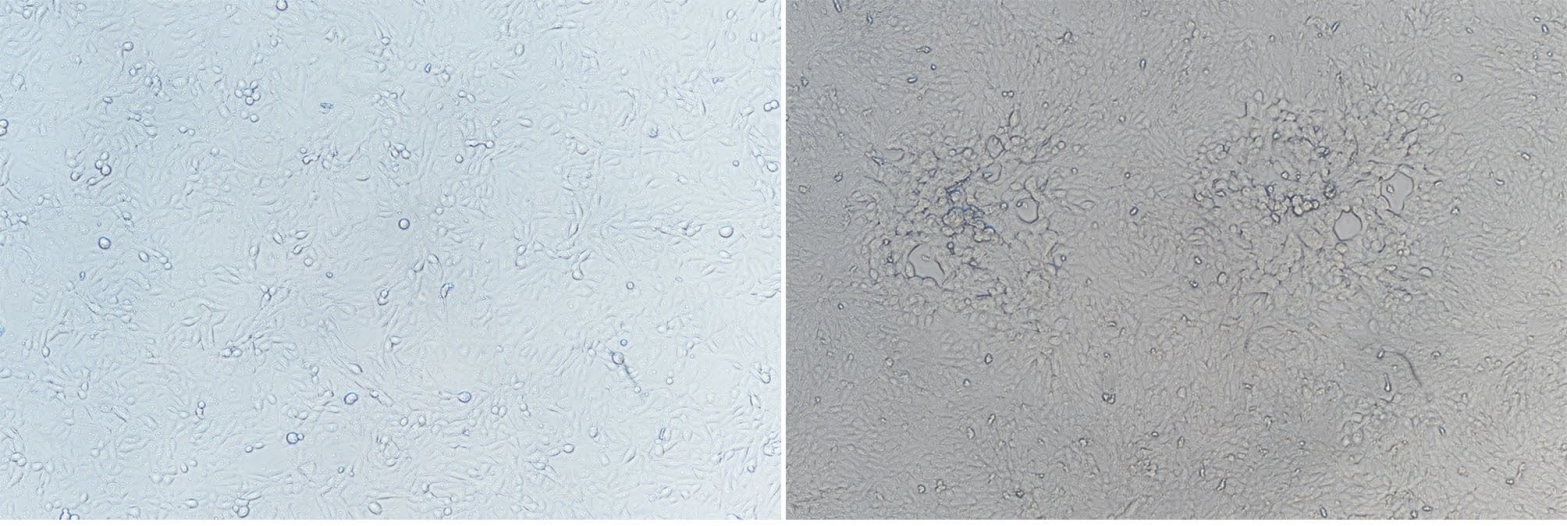
MDBK cells were grown for 72 h with nine passages. Cells were initially inoculated with the EHV-1-positive aborted fetal lung tissue lysate from donkeys (right panel) or left untreated (left panel)

**Fig. 5:**
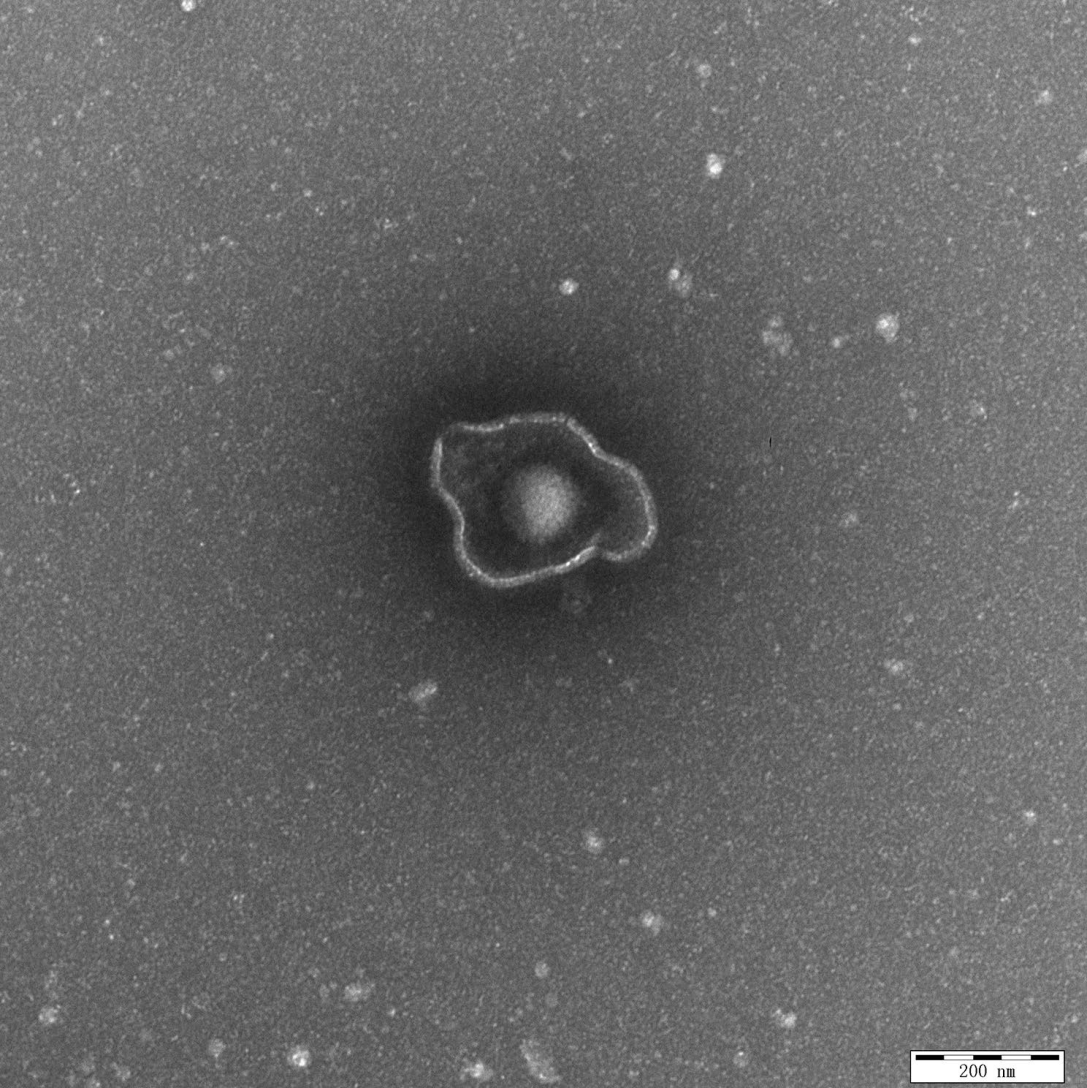
The virions of donkey EHV-1 Chabuchar/2020 display distinct morphologies

To further confirm the neutralization activity of the viral IgG-positive samples, serum-neutralization assays were conducted in MDBK cells using the 100 donkey sera. This showed that 72 of the 100 samples were able to neutralize 300 TCID_50_ (50% tissue-culture-infective dose) of EHV-1 at a dilution of 1:10 − 1:20.

Significant similarity was observed between the horse EHV-1 and donkey EHV-1 Chabuchar/2020 strains, with the complete ORF33 sequences of the two strains sharing a nucleotide similarity of 99.7 to 100% and an amino acid similarity of 99.5 to 100%. Furthermore, the identified sequence also aligned with reference strains of horse EHV-1 found in different countries, such as China (ZS-01: MZ561483, ZS-2: MZ561484, ZS-5: MZ561485, ZS-22: MZ561493, ZS-24: MZ561495, ZS-25: MZ561496, etc.), UK (Ab1: KU206468, Ab4: AY665713, and Army: KU206477), in Japan (NY03: KF644569, 01c1: KF644578), US (RacL11: MF975656), India (Meerut: MT077857), and Australia (717 A-82: KT324733, 438 − 77: KT324734). The results of both the DNA and amino acid alignments showed a 100% similarity level. These findings suggest that the ORF33 genes of donkey and horse EHV-1 display significant genetic conservation. The ORF33 nucleotide sequences were used to construct a phylogenetic tree, showing that the identified EHV-1 strains were grouped within cluster 1 of EHV-1 strains found in horses (Fig. 3).

To determine whether the newly identified donkey EHV-1 Chabuchar/2020 had neuropathogenic or non-neuropathogenic characteristics, a partial PCR amplification was conducted on the ORF30 gene (559 nucleotides) of EHV-1 Chabuchar/2020 using respective primers (Table S1). The results demonstrated that the partial ORF30 sequence of the neuropathogenic EHV-1 Chabuchar/2020 (ON624152) shared 100% nucleotide and amino acid sequence identity with the referenced strains of EHV-1 (OM047215-OM047257), thus suggesting a high degree of genetic conservation. Moreover, similar to the neuropathogenic EHV-1 strains ZS-01, ZS-2, ZS-5, ZS-8, ZS-10, ZS-11, ZS-16, and ZS-17 (OM047215-OM047222), the identified donkey EHV-1 strain had a G at position 2254 in the nucleotide sequence (resulting in an Asp residue at position 752 of the viral DNA polymerase) (Fig. 4). Therefore, the identified strain was confirmed to be a neuropathogenic EHV-1.

Furthermore, phylogenetic analysis of the ORF68 genes classifies all EHV-1 strains into VII groups that are closely related to strain origin [14, 16, 28]. The constructed phylogenetic tree of the ORF68 sequences showed the presence of the donkey EHV-1 identified in this study and the EHV-1 found in aborted fetuses of Yili mares in China (ON624153, ON637792-ON637805) on a distinct branch. They were classed as a new group, namely group VIII EHV-1 (Fig. 5). The results suggest that EHV-1, detected in aborted fetuses of both Yili mares and donkeys, may propagate independently in the Yili region of Northern Xinjiang, China.

## Discussion

The milk donkey sector is undergoing rapid development and contributes significantly to the economy of Xinjiang. However, abortion in donkey mares has a negative impact on the milk donkey industry. Wang et al. [29] found an association between EHV-8 and donkey abortions in Shangdong Province, China; however, the current study did not identify this association. EHV-1 is commonly linked to conditions such as respiratory complications and equine abortion [2–8]. In previous studies, Negussie et al. (2017) and Temesgen et al. (2021) identified the presence of EHV-1 in Ethiopian donkeys with and without respiratory diseases [9, 10]. The present study is the first to identify EHV-1 in samples of aborted donkey fetuses, suggesting that there is cause for concern regarding EHV-1 infection as a cause of donkey abortions, even though the virus has only been detected in donkeys from China. A recent study [8] found that EHV-1 was responsible for a significant number of abortion storms among Yili mares in Zhaosu County, and the majority of milk donkeys originate from this county. Therefore, the detection of EHV-1 in aborted samples obtained from an unvaccinated donkey is not unexpected. The prevalence of EHV-1 in China’s donkeys will be investigated further to confirm the link between this virus and diseases affecting donkeys.

Despite the initial identification of EHV-1 in nasopharyngeal samples from donkeys [9, 10], there is currently no published information regarding the genetic composition or viral particles of EHV-1 in donkeys. In the present study, the donkey EHV-1 Chabuchar/2020 strain was successfully isolated and analyzed (Fig. 2), and the partial ORF33 sequences of three donkey EHV-1 were sequenced and submitted to GenBank (ON809533-ON809535).

To date, there have been no serological investigations of EHV-1 in donkeys. In the present study, 72 of the 100 aborted donkey fetuses were positive for EHV-1 IgG, suggesting that EHV-1 was a potential cause of the abortion outbreak among donkeys in North Xinjiang, China, in 2020. Further research will be conducted on the serological epidemiology of EHV-1 in donkey populations in Xinjiang.

The current study compared the sequences of ORF33, ORF30, and ORF68 of the EHV-1 Chabuchar/2020 strain and horse EHV-1 reference strains from abortion samples of Yili mares at the Chinese State Studs of Zhaosu, China. The sequences (MZ561483-MZ561485, MZ561493, MZ561495-MZ561497, MZ561500-MZ561503, MZ561507-MZ561510, MZ561513, MZ561515, MZ561517, MZ561518, MZ561521-MZ561523, MZ561525, OM047215-OM047257, ON637792-ON637805) showed 100% nucleotide and amino acid identities. Furthermore, the donkey EHV-1 strain responsible for abortions showed neuropathogenic characteristics, thus aligning with the EHV-1 strains responsible for abortions in Yili mares. Moreover, based on the phylogenetic analysis of ORF68 sequences, it was found that both the donkey and Yili mare EHV-1 strains of group VIII originated from the Yili area in Xinjiang. Thus, it can be concluded that the EHV-1 Chabuchar/2020 strain may have originated in Yili horses.

## Conclusions

In conclusion, this study is the first to identify EHV-1 in aborted donkey fetuses. The findings suggest that EHV-1 may have been the causative agent of donkey abortions. Further analysis also showed evidence suggesting a potential involvement of the neuropathogenic strain of EHV-1 found in the new group VIII. The results suggest the importance of awareness of the involvement of EHV-1 in donkey abortions and also encourage further research on EHV-1 in donkeys and the development of vaccines targeting the virus.

### Electronic Supplementary Material

Below is the link to the electronic supplementary material.

Supplementary Material 1

## Data Availability

No datasets were generated or analysed during the current study.

## Abbreviations

EHV-1: Equine herpesvirus type 1
ORF: Open reading frame
G: Guanidine
A: Adenine
N: Asparagine
D: Aspartic acid
