# Supplementary material for: First identification and isolation of equine herpesvirus type 1 in aborted fetal lung tissues of donkeys
Source: Virol J. 2024 May 27;21:117. doi: 10.1186/s12985-024-02390-2 (PMC11131334; doi:10.1186/s12985-024-02390-2)
Supplement: Supplementary file 1 — Supplementary Material 1 [file 12985_2024_2390_MOESM1_ESM.docx]

**Table S1** Primer sequences used in this study

| Primer | Sequence (5′-3′) | Product size (bp) |
| --- | --- | --- |
| EHV1 ORF68-F | CTGTGGGTGGATGAACTAATG | 500 |
| EHV1 ORF68-R | CATCGAGTTTATTGACCCTACC |  |
| EHV1 complete ORF33-F1 | ATGTCCTCTGGTTGCCGTTCTG | 1644 |
| EHV1 complete ORF33-R1 | CTCGTTTGTGCAAGCGATGTAG |  |
| EHV1 complete ORF33-F2 | GTGAGATCTAACCGCACCTACGAC | 1463 |
| EHV1 complete ORF33-R2 | TTAAACCATTTTTTCATYTTCCATG |  |
| EHV1 partial ORF30-F | CGGAGTAAGGCTTGTGGTTTCG | 559 |
| EHV1 partial ORF30-R | GTGGGCTACCAGGGAGCAAAG |  |
| EHV1 ORF33-F | GGGTCCTACAGATTTACTATTCG | 592  587  881  716  316 |
| EHV1 ORF33-R  EHV-4 ORF33-F  EHV-4 ORF33-R  EHV-5 ORF8-F  EHV-5 ORF8-R  EHV-2 ORF8-F  EHV-2 ORF8-R  EHV-8 G1-F  EHV-8 G1-R | TACACCACGCAGTTGCTATTCTAC  TGGGTCCTACAGATTTACTATTCG  TACACCACGCAGTTGCTATCCTAC  TGTATCACCGTGGACCAAGAGAG  TCAAAGATGGATCTTCTCTGAGTG  GGTGACACTATAGAGATGTCRCC  CTGTTGATGCTCTTTCTGAGATTG  TCAGACTGTCACTCGTGGGA  CCTGGAGGCCGTTTAACACA |  |
|  |  |  |
|  |  |  |
